# EEG Parameter Selection Reflecting the Characteristics of Internet Gaming Disorder While Playing League of Legends

**DOI:** 10.3390/s23031659

**Published:** 2023-02-02

**Authors:** Jung-Yong Kim, Dong-Joon Kim, Sung-Kyun Im, Hea-Sol Kim, Ji-Soo Park

**Affiliations:** 1 Department of HCI, Hanyang University ERICA, Ansan-si 15588, Republic of Korea; 2 Department of Industrial and Management Engineering, Hanyang University ERICA, Ansan-si 15588, Republic of Korea; 3 Department of Industrial Engineering, Hanyang University, Seoul 04763, Republic of Korea

**Keywords:** internet gaming disorder, EEG parameter, in game, statistical process, key brain region, crossing phenomenon

## Abstract

Game playing is an accessible leisure activity. Recently, the World Health Organization officially included gaming disorder in the ICD-11, and studies using several bio-signals were conducted to quantitatively determine this. However, most EEG studies regarding internet gaming disorder (IGD) were conducted in the resting state, and the outcomes appeared to be too inconsistent to identify a general trend. Therefore, this study aimed to use a series of statistical processes with all the existing EEG parameters until the most effective ones to identify the difference between IGD subjects IGD and healthy subjects was determined. Thirty subjects were grouped into IGD (n = 15) and healthy (n = 15) subjects by using the Young’s internet addition test (IAT) and the compulsive internet use scale (CIUS). EEG data for 16 channels were collected while the subjects played League of Legends. For the exhaustive search of parameters, 240 parameters were tested in terms of t-test, factor analysis, Pearson correlation, and finally logistic regression analysis. After a series of statistical processes, the parameters from Alpha, sensory motor rhythm (SMR), and MidBeta ranging from the Fp1, C3, C4, and O1 channels were found to be best indicators of IGD symptoms. The accuracy of diagnosis was computed as 63.5–73.1% before cross-validation. The most interesting finding of the study was the dynamics of EEG relative power in the 10–20 Hz band. This EEG crossing phenomenon between IGD and healthy subjects may explain why previous research showed inconsistent outcomes. The outcome of this study could be the referential guide for further investigation to quantitatively assess IGD symptoms.

## 1. Introduction

Online games have become a major platform of digital entertainment. People are easily exposed to games, especially after social distancing forced them to stay for long hours at home due to COVID-19 [1]. Therefore, the risk of indulgence in online games has become a serious social health issue.

In 2018, the World Health Organization (WHO) officially included gaming disorder in the International Classification of Disease (ICD)-11 for the benefit of people suffering from disorders who need medical attention as well as to prevent disease due to the excessive use of games. However, despite the WHO’s attempt to enhance public health in this regard, gaming disorder has been stigmatized as an addictive behavior similar to gambling addiction, which is perceived as anti-social behavior. Lee [2] stated that gaming disorder should be understood in terms of maladaptation or sickness rather than in terms of being right or wrong. Aarseth et al. [3] urged the removal of the ICD-11 proposal to avoid causing significant stigma to children and adolescents who play video games as a leisure activity as part of a healthy lifestyle. Thus, it became challenging to correctly diagnose gaming disorder without misidentifying healthy game players as game addicts.

A questionnaire was employed to clinically categorize the gaming disorder group. To identify the gaming disorder group, Young [4] developed a scale, the internet addiction test (IAT), while Meerkerk et al. [5] developed the compulsive internet use scale (CIUS). The reliability of these surveys has been validated when it comes to categorically assessing the risk of internet addiction or compulsive use. However, subjective questionnaires have a limitation in individually specifying the level of disorder to decide whether the subject needs serious medical attention.

To identify the level of internet game disorder (IGD), various efforts have been made to quantify the individual status at resting state electroencephalogram (EEG). Lee et al. [6] also measured the resting-state EEG for the internet addiction group with and without depression. The absolute power of the delta and beta decreased in the internet addiction group without depression. However, for the group with depression, an increased relative theta power and decreased relative alpha power were observed. However, a previous study reported contradictory results in terms of delta power. Kim et al. [7] compared 20 patients with IGD with healthy subjects for 6 months. In this study, patients with IGD showed increased delta and theta power activities in the resting state. After 6 months of treatment, the increase in delta band activity normalized, which was significantly correlated with a decrease in IGD symptoms.

In addition, there have been studies on beta power decreases in the IGD group. Choi et al. [8] measured the resting-state EEG of an internet addiction group. They reported that high impulsivity and impaired inhibitory control were observed in the internet addicted group. Moreover, it was said that there was a decrease in the absolute power of the beta band and an increase in the absolute power of the gamma band in the internet addiction group. They also mentioned that these differences were related to impulsivity in the internet addiction group. This result corresponds to the outcome of Lee et al. [6]. However, there was a previous study that reported increased beta power. Rabadanova and Taygibova [9] investigated the EEG patterns of subjects with non-chemical addictions, such as gaming and internet addiction. They showed that the EEG shifted to a high-amplitude wave, including beta1 (13.0–19.9 Hz) and beta2 (20.0–35.0 Hz), in terms of the manifestation index (%) when game dependence was observed.

These contradictory results have also been observed in the alpha power domain. Wang and Griskova-Bulanova [10] investigated the association between resting prefrontal alpha rhythm and internet use by EEG in a non-clinical population. This study indicated a positive correlation between the alpha power of the frontal lobe and scores on the internet addiction test when the eyes were closed. However, Son et al. [11] found that the absolute alpha power of the gaming disorder group was lower than that of the other groups when they compared the resting state of EEG among subjects with gaming disorder, alcohol dependency, and healthy subjects.

As summarized in Table 1, the above results are too inconsistent to identify a common trend for IGD in relation to EEG measurement during a resting state. This is likely due to the variability of EEG response patterns among the IGD group in the resting state, and other studies were initiated to measure the EEG while subjects were playing real games. Hafeez et al. [12] recorded EEG measurements during mobile gaming to identify the different wave patterns between game addicts and non-addicts. They used cluster analysis and cross-correlation analysis to mathematically define the significantly different signal patterns of θ and θ/σ in the right occipital lobe. They reported an 86.9% (±9.49) detection rate in terms of the addicted group and an 88.0% (±10.41) detection rate in terms of the non-addicted group based on a θ parameter measurement at O2 (right occipital region). Kim et al. [13] used heart rate variability (HRV) parameters to examine the patterns of players with gaming disorders during internet game playing. They used the IAT and CIUS tests to group the subjects. The time series and frequency-based HRV parameters were thoroughly tested to determine the most effective parameters to differentiate the groups. They identified the following key parameters: the root mean square of successive differences (RMSSD) between normal heartbeats, the frequency activity (LF) at the 0.04–0.15 Hz, and the proportion of successive interval differences (pNNI20) ≤ 20 ms within all intervals. The logistic regression model indicated a nearly 70% accuracy when distinguishing the two groups. These studies demonstrate the potential statistical power of using quantifiable parameters, although the accuracy needs to be further improved after cross-validation.

Therefore, in this study, we used a different experimental setting. That is, the EEG sign of IGD among the subjects was measured when they were actively exposed to gaming events. In particular, the authors performed a series of statistical processes to determine the critical parameters and brain region genuinely reflecting the characteristics of IGD among subjects. This study also used a logistic regression model to quantitatively differentiate between the IGD and healthy groups to find the most effective EEG parameters in identifying IGD characteristics.

This paper consists of methods, results, discussions, and conclusions. In the method chapter, the process of recruiting subjects, apparatus setting, experimental design, and statistical analysis methods are described. In the results, it is shown how the effective parameters were selected through t-test, factor analysis, Pearson correlation coefficient, and logistic regression analysis. Importantly, the crossing phenomenon, which was newly observed in this study, is emphasized. The performance of parameters is finally reported. In the discussion, the critical findings of the study are outlined and the conclusions are summarized.

## 2. Methods

### 2.1. Subject Grouping and Control

Concerning the two groups for the study, the IGD and healthy groups were determined based on both the IAT (Table A1 in Appendix A) by Young and De Abreu [14] and the CIUS test (Table A2) by Meerkerk et al. [5]. The subjects with scores of ≥50 in the IAT and a mean ≥2.5 in the CIUS test were categorized as the IGD group. Young [4] initially suggested that IAT scores between 40 and 69 signify problems due to internet use. On the other hand, Peng et al. [15] used ≥40 points to identify IGD individuals when they spent ≥4 h per day and ≥30 h per week on internet gaming. Many researchers have employed the CIUS score to group gaming disorders [16,17,18]. After self-reported scoring, 15 subjects each in the IGD and healthy groups were recruited for the experiment. Subjects had a half day off for the 3 h experiment to prevent accumulated fatigue from working. They were asked not to consume any caffeine and alcohol on that day, and smoking was prohibited for 2 h before the experiment. They played the game at the skill level they were used to playing to maintain a consistent intensity. Each game lasted about half an hour, with a 10 min break, including warming-up practice. Two game trials were executed for data collection. No physical fatigue was observed. Subjects were continuously monitored by experimenter, and all the instructions for the subjects were standardized. The experiment was stopped if any discomfort was reported by subjects. All the subjects were compensated at the end of experiment. All the participating subjects submitted their demographic information when they were filling in the addiction questionnaire. The age of healthy subjects and IGD subjects was 24.3 (±2.74) and 22.7 (±2.20), respectively. Since the subjects were recruited from a relatively homogenous student community, the demographic characteristics were very similar each other. The gaming experience of all subjects was longer than 2 years. Regarding the skill level of subjects, the healthy subjects ranged from “Bronze” tier to “Platinum” tier, and the majority were “Silver” tier. Regarding the IGD subjects, they ranged from “Bronze” tier to “Platinum” tier, and the majority were “Gold” Tier. Subjects without a history of diagnosis of mental disorders such as depression and ADHD were included in the experiment. At the end of measurement, the subjects were compensated for the experiment.

### 2.2. Apparatus

**Figure 1:**
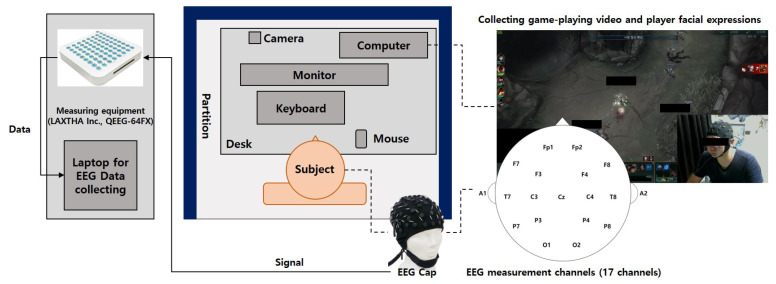
Experimental setting.

League of Legends (LOL) from Riot Games Inc. (Los Angeles, CA, USA) was used for the experiment. This game is known to be the most frequently played game among internet game players [19]. For data acquisition, 16 channels of *QEEG-64FX* (LAXTHA Inc., Daejeon, Republic of Korea) were used for the EEG measurements (Figure 1). The electrode attachment sites were selected using the international 10–20 system that was used in previous studies [6,11]. An additional Cz channel was used as a time marker to track the first screen of the game during the experiment. A data collection program called *Telescan* (LAXTHA, Inc.) was used. The data-sampling rate was set to 500 Hz. The experiment was conducted in a room equipped with a computer, desk, and chair, in a space where other external stimuli were suppressed.

### 2.3. Experimental Design

The experiment was designed to test the null hypothesis: EEG parameters showed no significant differences between subjects with IGD and healthy subjects. Therefore, a between-subject design was employed in this study. The independent variable was set as the addiction status of the group determined by the questionnaire score. For dependent variables, 15 EEG parameters, including 6 frequency domain parameters and 9 additional composite parameters, were used: the relative power of the Theta, Alpha, Beta, sensorimotor rhythm (SMR), MidBeta, HighBeta, Theta/Beta, Beta/Theta, Alpha/MidBeta, Theta/MidBeta, MidBeta/Alpha, Alpha/Theta, (SMR+MidBeta)/Theta, Beta/Alpha, and MidBeta/Theta [20,21,22,23,24,25]. These parameters were measured in 16 lobes of the brain, resulting in a total of 240 dependent parameters in this study. Insignificant dependent parameters were eliminated in each step of the statistical analysis to build logistic regression models in this study.

### 2.4. Procedure

**Figure 2:**
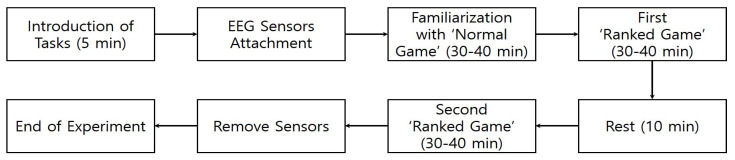
Experimental process.

EEG electrodes were placed according to a 10–20 system. Seventeen channels were used in this experiment. The measurement channels were Fp1, Fp2, F3, F4, C3, C4, P3, P4, O1, O2, F7, F8, T7, T8, P7, P8, and Cz, with two reference electrodes (A1 for the left ear and A2 for the right ear) and one ground electrode placed on the 7th cervical vertebra. After explaining the experimental procedure to the subjects, the EEG sensor cap was mounted, and the signal stability was checked for 1 min while they were relaxing. To familiarize the subject with the environment, they played a ‘normal game’ prior to the actual EEG measurement. Then, the subjects played a ‘ranked game’ twice with a 10-min break between games. EEG data were collected during the game time, and the data analysis for all parameters was performed based on the data obtained at 30–40 min. The subjects were not informed about their questionnaire scores. Therefore, they did not know which group they belonged to. The detailed experimental procedure is illustrated in Figure 2.

### 2.5. Data Analysis

**Figure 3:**
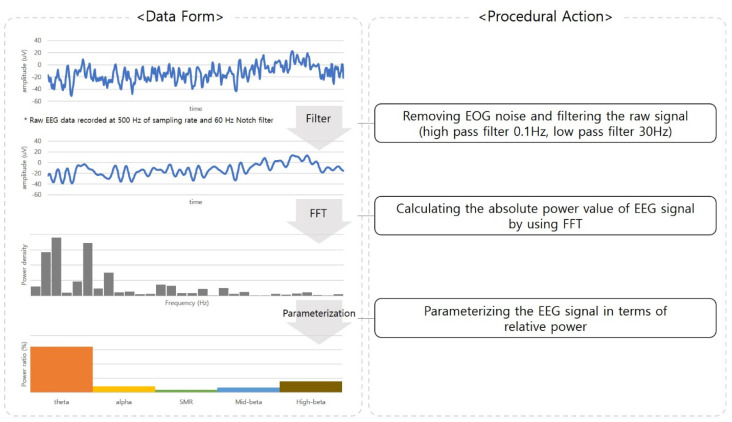
Preprocessing of the EEG data.

To preprocess the EEG data, the *Telescan* software provided by the manufacturer was used. The collected raw EEG data were converted into a frequency band through fast Fourier transform (FFT) with a Hamming window. The FFT window size of the EEG was set to 20 min of actively playing games. The absolute power values of each frequency range were calculated. The frequency ranges were specified as follows: Theta 4–8 Hz, Alpha 8–12 Hz, SMR 12–15 Hz, MidBeta 15–20 Hz, HighBeta 20–30 Hz, and Beta 12–30 Hz. Because the absolute power value could be affected by the subject’s scalp thickness, skull thickness, and the skin contact quality of the electrodes, causing a random error, the relative power value was used in this study. The relative power value is presented as the ratio of the absolute power of a frequency band to that of the entire frequency band. The relative power is a ratio value with no units and is represented between 0 and 1. The process of preprocessing of the EEG data is illustrated in Figure 3.

**Figure 4:**
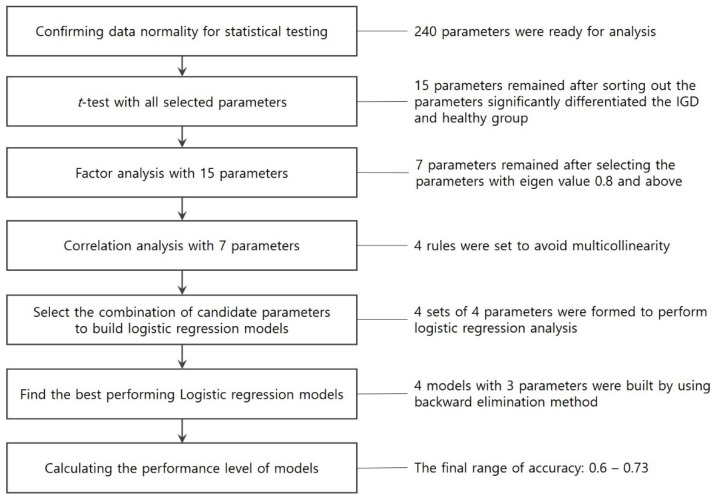
Parameter elimination process.

An exhaustive search for the optimal combination of model parameters was performed with a total of 240 parameters (15 variables × 16 channels) using SPSS (version 26). First, the Kolmogorov–Smirnov normality test and *t*-test were performed to determine the parameters that significantly differentiated the two groups in every lobe of the brain. Fifteen parameters were selected for the process. Second, factor analysis was performed to determine the main components and eigenvalues of the individual parameters that best represented the discriminant features of the EEG signals. The four factors were sorted based on eigenvalues > 1.0. Parameters with eigenvalues of 0.8 and above were selected as candidate parameters for the statistical model building. Seven parameters were selected for this process. Third, a correlation analysis was performed to examine the statistical redundancy among the seven parameters to enhance the discriminant power of the logistic regression model. Thus, four sets of four parameters with the least influence in terms of multicollinearity were selected. Fourth, a backward elimination technique was used to determine insignificant parameters in the candidate models. Finally, four models were suggested, and their sensitivity, specificity, and accuracy values were reported. Figure 4 is an illustration of the parameter elimination process described above.

### 2.6. Ethics

The experiment was conducted in accordance with the Declaration of Helsinki and regulations under consideration of the Institutional Review Board of Hanyang University in the Republic of Korea (IRB approval number: HYU-2019-08-004-1). All participants understood the study procedure and provided written informed consent before participation.

## 3. Results

### 3.1. t-Test Results

A t-test was performed using the individual EEG and composite variables. A total of 240 parameters were tested, and the 15 parameters that showed significant results (*p* < 0.1) are shown in Table 2. Each individual parameter was independently treated for the t-test.

### 3.2. Factor Analysis

A factor analysis was performed using 15 parameters. As a result of the factor analysis, four components were extracted, and eigenvalues were calculated. The results are shown in Table 3.

The seven parameters with eigenvalues of 0.8 and above were selected and are presented in Table 4.

### 3.3. Correlation Analysis to Avoid Multicollinearity

The correlation coefficients of the seven parameters are listed in Table 5. Through this correlation analysis, the multicollinearity among the selected parameters was examined. Parameters with correlation coefficient values greater than the absolute value of 0.6 were not coupled together to avoid multicollinearity for model development.

To avoid multicollinearity in developing the logistic regression model, the following parameter couples were not used in the model: R.MidBeta (C3) and MidBeta/Alpha (C3) (*ρ* = 0.848), MidBeta/Alpha (C3) and Alpha/MidBeta (C3) (*ρ* = −0.846), R.MidBeta (C3) and Alpha/MidBeta (C3) (*ρ* = −0.752), and Alpha/MidBeta (C3) and R.Alpha (C4) (*ρ* = −0.752).

Finally, four sets of four candidate parameters were prepared for logistic regression analysis to discriminate between the healthy and IGD groups. The final candidate parameters of each model are shown in Table 6.

### 3.4. Logistic Regression Analysis

**Figure 5:**
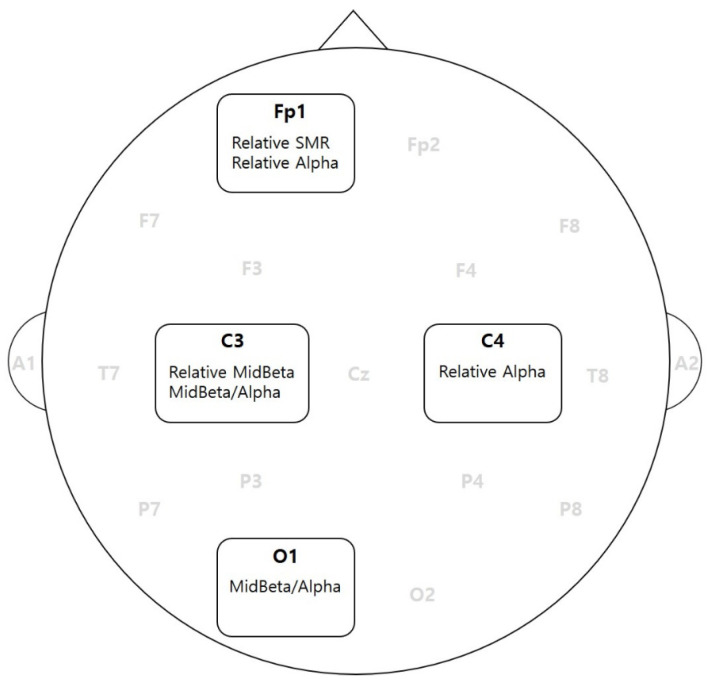
Location of EEG parameters significantly differentiating the IGD group.

A logistic regression was used to find the best combination of parameters in terms of calculating the best probability of accuracy. A backward elimination method was used to find the best set of logistic models. The final models and accuracy values are listed in Table 7. The equations used for the models are listed in Table 8. The final accuracy range was 63.5% to 73.1%. The final parameters and brain regions are shown in Figure 5.

### 3.5. Crossing Phenomenon at a Particular Frequency Range

A reversal pattern in terms of EEG was observed between subjects with IGD and healthy subjects in the SMR and MidBeta ranges of the EEG. This phenomenon can be referred to as the EEG crossing phenomenon, that is, the relative power of the EEG among IGD subjects became weaker than that of healthy subjects in the frequency range between SMR and MidBeta. This phenomenon was observed in four lobes: Fp1, C3, C4, and O1. The observed critical frequency range was approximately 10–20 Hz.

## 4. Discussion

### 4.1. Resting State vs. Dynamic Gaming State

There is no standardized testing protocol to identify IGD subjects. In the literature, most studies were conducted during a resting state and therefore had the advantage of collecting a stable signal from a statically positioned subject in a relatively well-controlled experimental setting; however, taken together they do not provide an agreeable conclusion. Therefore, looking into the brain signal during the action of game playing was an inevitable step, despite the difficulty in controlling subjects during the experiment. Hafeez et al. [12] and Kim et al. [13] examined the bio-signals of IGD subjects during game playing and found significantly different EEG and ECG patterns in the IGD group compared to the healthy group. Moreover, the current study showed a 63.5%–73.1% accuracy in identifying the IGD subjects. This was not a sufficient level of accuracy for diagnostic purposes. The current outcome should be further examined by using various experimental conditions and extended subject pools.

### 4.2. Limitation of Subject Categorization

The self-report survey could serve the purpose of categorizing IGD subjects (or potential IGD subjects) and healthy subjects. To minimize type I errors, this study employed both the IAT and CIUS for subject grouping, and only subjects who satisfied both classifications were allowed to participate in the experiment. However, despite such efforts, the IGD subjects could not be confirmed as game addicted patients who required immediate medical attention. This limitation in terms of the categorization of IGD subjects exists as long as surveys are used without medical diagnosis. This could be the source of latent experimental errors, which may have lowered the accuracy of the study. Therefore, further collaboration with medical professionals is expected.

### 4.3. Controlling the Level of Immersion of Game Players

The experimenter could not control the participants’ thoughts. It was only possible to ask the subjects to focus on the task, but it was difficult to ensure that they were willingly conducting the experiment. Therefore, in this study, subjects were directed to play the game using their own game account and keep the records of wins and losses so that they could strongly immerse themselves to obtain a high score in the game. Simultaneously, the League of Legends game itself has a function to match the player with the same tier group, which makes the players feel competitive during game playing. The authors took advantage of this function to maintain the highest level of immersion for the participating subjects.

### 4.4. EEG Crossing Phenomenon EEG Characterizing the IGD

**Figure 6:**
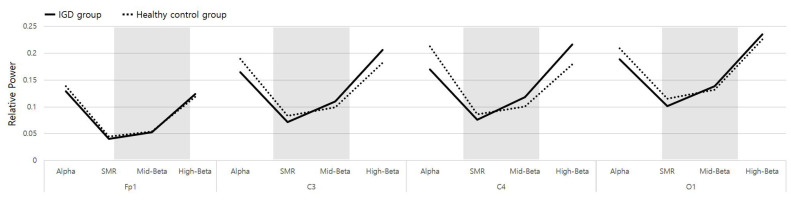
Crossing phenomenon was observed (shading area) and presented at four lobes when Alpha was at 8–12 Hz, SMR at 12–15 Hz, MidBeta at 15–20 Hz, and HighBeta at 20–30 Hz.

The IGD subjects showed a lower relative power value than the healthy subjects in the Alpha and SMR band, and they showed a higher relative power value in the MidBeta and HighBeta bands. This ‘crossing phenomenon’ is presented in Figure 6. This result of this study is intriguing. That is, the beta relative power value can be higher or lower depending on which frequency of the beta band is analyzed. In fact, in previous studies, the beta value was either higher or lower among IGD subjects. Therefore, it can be said that the crossing phenomenon might be the cause of inconsistent beta outcomes. The range of the crossing phenomenon was located between the SMR band (12–15 Hz) and the MidBeta band (15–20 Hz). This EEG band needs to be closely investigated in future studies to determine any signature pattern in terms of IGD subjects, if it exists. This also suggests that EEG frequency analysis for IGD should be performed with a detailed frequency index including the SMR and MidBeta range.

### 4.5. Key Location in the Brain

The current study also found that Fp1 was a critical location to observe EEG information to identify the IGD symptoms, with this agreeing with many studies. Moreover, C3, C4, and O1 were found to be important locations to see the signs of IGD symptoms during game playing. C3 and C4 had a marginal correlation coefficient (0.686), and C3 alone could contribute to make the best logistic regression, as was shown in model 4. That is, the odd number locations were relatively sensitive sites of the lobe. This showed that the right and left lobes respond differently. This also implies that the desynchronization of EEG signals can be an important source for identifying the signal pattern of IGD [26,27,28].

### 4.6. Exhaustive and Continuing Search for the Best Model

All 240 EEG parameters were tested in this study. Multiple steps of the elimination process were designed by using four steps of statistical operations. This was performed to determine the most effective parameters. The current methodology includes tedious data pre-processing, a series of statistical computations, and the model building technique, all of which were laborious. However, these steps were necessary since no prior attempt has made using such a thorough bottom-up selection process to identify IGD characteristics during game playing. The current logistic regression model was used for finding the best parameters. However, the final diagnostic model targeting an accuracy level of 80% and above needs to be further developed for future study.

## 5. Conclusions

This study demonstrated the process to objectively select the most effective EEG parameters to identify IGD characteristics during game playing. The results of the study could be a referential guide for future research to identify IGD symptoms for diagnosis. A series of statistical analyses were performed, and this statistical operation can be referenced for analyzing EEG data. This study also revealed that Fp1, C3, and O1 were important locations to observe the IGD features, and MidBeta/Alpha (C3), R.SMR (Fp1), and MidBeta/Alpha (O1) were found to be the most effective parameters. A unique crossing phenomenon concerning the relative EEG power at the SMR and MidBeta range was observed. This relative power inversion between IGD subjects and healthy subjects should be further investigated since it could provide the reason for previous inconsistent findings in the Beta band range. The exhaustive steps of the parameter elimination process could provide an unbiased picture of IGD subjects’ characteristics, particularly during game playing. The current logistic regression model needs to be further improved by cross validation. The diagnostic power of the model could be bettered in conjunction with another bio-signal in future study.

## Figures and Tables

**Table 1:** Summary of various EEG outcomes associated with presumably IGD subjects. All results were tested during resting state.

Studies	Observed Contradictory Signs of IGD
Lee et al. [6]	Decrease in absolute power of the delta and beta without depression; increase in relative theta power and decrease in relative alpha power with depression
Kim et al. [7]	Increased activity of slow-wave (delta and theta) EEG
Choi et al. [8]	Decrease in the absolute power of the beta band and increase in the absolute power of the gamma band
Rabadanova and Taygibova [9]	EEG shifted to a high-amplitude wave (beta power domain)
Wang and Griskova- Bulanova [10]	Positive correlation between the alpha power of frontal lobe and internet addiction test scores
Son et al. [11]	A relatively low absolute alpha power

EEG, electroencephalogram; IGD, internet gaming disorder.

**Table 2:** Fifteen parameters with significant *t*-test results (R = relative power).

Parameter	t-Value	*p*-Value	Difference of Mean
R.Alpha (Fp1)	−2.296	0.026	−0.01451
R.Alpha (F4)	−2.034	0.047	−0.02014
R.Alpha (C4)	−2.549	0.014	−0.04501
R.Alpha (O2)	−1.893	0.064	−0.01973
R.SMR (Fp1)	−2.367	0.022	−0.00763
R.SMR (F3)	−2.171	0.035	−0.02230
R.MidBeta (C3)	1.788	0.080	0.01341
R.MidBeta (P3)	2.268	0.028	0.01185
Alpha/MidBeta (F4)	−1.935	0.059	−0.47890
Alpha/MidBeta (C3)	−2.509	0.015	−0.48591
Alpha/Theta (F3)	−1.843	0.071	−0.04283
MidBeta/Alpha (C3)	2.218	0.031	0.16886
MidBeta/Alpha (O1)	2.287	0.026	0.11124
MidBeta/Alpha (O2)	2.283	0.027	0.09449

**Table 3:** Components and eigenvalues of 15 parameters.

	Component			
	1	2	3	4
R.MidBeta (C3)	0.946			
MidBeta/Alpha (C3)	0.904			
Alpha/MidBeta (C3)	−0.816			
R.MidBeta (P3)	0.747			
Alpha/MidBeta (F4)	−0.502			
R.Alpha (C4)		0.839		
R.Alpha (F4)		0.794		
R.Alpha (O2)		0.742		
Alpha/Theta (F3)		0.629		
R.SMR (Fp1)			0.842	
R.Alpha (Fp1)			0.802	
R.SMR (F3)			0.767	
MidBeta/Alpha (O1)				0.843
MidBeta/Alpha (O2)				0.793

**Table 4:** Seven parameters representing the main components with eigenvalues of 0.8 and above.

Component 1	Component 2	Component 3	Component 4
R. MidBeta (C3)	R.Alpha (C4)	R.SMR (Fp1)	MidBeta/Alpha (O1)
MidBeta/Alpha (C3)		R.Alpha (Fp1)	
Alpha/MidBeta (C3)			

**Table 5:** Correlation coefficients of seven parameters.

		Comp. 1			Comp. 2	Comp. 3		Comp. 4
		R.MidBeta (C3)	MidBeta /Alpha (C3)	Alpha /MidBeta (C3)	R.Alpha (C4)	R.Alpha (Fp1)	R.SMR (Fp1)	MidBeta /Alpha (O1)
R.MidBeta (C3)	Coef.	1.000	0.848	−0.752	−0.163	−0.007	0.321	0.161
	*p*-value	0.000	0.000	0.000	0.125	0.482	0.010	0.127
MidBeta /Alpha (C3)	Coef.		1.000	−0.846	−0.403	−0.130	0.147	0.134
	*p*-value		0.000	0.000	0.002	0.180	0.150	0.172
Alpha /MidBeta (C3)	Coef.			1.000	0.686	0.209	−0.121	−0.416
	*p*-value			0.000	0.000	0.069	0.196	0.001
R.Alpha (C4)	Coef.				1.000	0.364	0.223	−0.407
	*p*-value				0.000	0.004	0.056	0.001
R.Alpha (Fp1)	Coef.					1.000	0.664	−0.254
	*p*-value					0.000	0.000	0.034
R.SMR (Fp1)	Coef.						1.000	0.024
	*p*-value						0.000	0.433
MidBeta /Alpha (O1)	Coef.							1.000
	*p*-value							0.000

**Table 6:** Four sets of parameters were selected without violating multicollinearity.

Model Candidates No.	Final Candidate Parameters
1	R.MidBeta (C3), R.Alpha (C4), R.Alpha (Fp1), MidBeta/Alpha (O1)
2	MidBeta/Alpha (C3), R.Alpha (C4), R.Alpha (Fp1), MidBeta/Alpha (O1)
3	R.MidBeta (C3), R.Alpha (C4), R.SMR (Fp1), MidBeta/Alpha (O1)
4	MidBeta/Alpha (C3), R.Alpha (C4), R.SMR (Fp1), MidBeta/Alpha (O1)

**Table 7:** Results of logistic regression analysis.

Model No.	Model Variable	B Value	*p*-Value	Sensitivity	Specificity	Accuracy
1	R.MidBeta (C3)	21.180	0.087	0.692	0.731	71.2%
	R.Alpha (C4)	−13.835	0.093			
	R.Alpha (Fp1)	−24.077	0.097			
2	MidBeta/Alpha (C3)	2.396	0.077	0.577	0.692	63.5%
	R.Alpha (Fp1)	−25.345	0.079			
	MidBeta/Alpha (O1)	3.383	0.099			
3	R.MidBeta (C3)	31.525	0.020	0.692	0.731	71.2%
	R.SMR (Fp1)	−94.126	0.004			
	MidBeta/Alpha (O1)	4.589	0.045			
4	MidBeta/Alpha (C3)	3.218	0.026	0.731	0.731	73.1%
	R.SMR (Fp1)	−84.173	0.007			
	MidBeta/Alpha (O1)	4.888	0.037			

**Table 8:** Final logistic regression models.

Model No.	Equation
(1)	$\ln\left(\frac{P}{1-P}\right)=3.317+21.180\times R.{\mathrm{MidBeta}}_{C3}-13.835\times R.{\mathrm{Alpha}}_{C4}-24.077\times R.Alpha_{Fp1}$
(2)	$\ln\left(\frac{P}{1-P}\right)=-0.883+2.396\times \frac{MidBeta_{C3}}{Alpha_{C3}}-25.345\times R.{\mathrm{Alpha}}_{\mathrm{Fp}1}+3.383\times \frac{{\mathrm{MidBeta}}_{O1}}{{\mathrm{Alpha}}_{O1}}$
(3)	$\ln\left(\frac{P}{1-P}\right)=-3.234+31.525\times R.MidBeta_{C3}-94.126\times R.SMR_{Fp1}+4.589\times \frac{MidBeta_{O1}}{Alpha_{O1}}$
(4)	$\ln\left(\frac{P}{1-P}\right)=-2.562+3.218\times \frac{MidBeta_{C3}}{Alpha_{C3}}-84.173\times R.SMR_{Fp1}+4.888\times \frac{MidBeta_{O1}}{Alpha_{O1}}$

## Data Availability

Not applicable.

## Used Questionnaires in the Experimental Setup

**Table A1:** Young’s internet addiction test (IAT).

Item	How Often...	Score
Q1	Do you find that you stay online longer than you intended?	
Q2	Do you neglect household chores to spend more time online?	
Q3	Do you prefer the excitement of the internet to intimacy with your partner?	
Q4	Do you form new relationships with fellow online users?	
Q5	Do others in your life complain to you about the amount of time you spend online?	
Q6	Do your grades or schoolwork suffer because of the amount of time you spend online?	
Q7	Do you check your email before something else that you need to do?	
Q8	Does your job performance or productivity suffer because of the internet?	
Q9	Do you become defensive or secretive when anyone asks you what you do online?	
Q10	Do you block out disturbing thoughts about your life with soothing thoughts of the internet?	
Q11	Do you find that you find yourself anticipating when you will go online again?	
Q12	Do you fear that life without the internet would be boring, empty, or joyless?	
Q13	Do you snap, yell, or act annoyed if someone bothers you while you are online?	
Q14	Do you lose sleep due to late night logins?	
Q15	Do you feel preoccupied with the internet when offline or fantasize about being online?	
Q16	Do you find yourself saying “just a few more minutes” when online?	
Q17	Do you try to cut down the amount of time you spend online?	
Q18	Do you try to hide how long you’ve been online?	
Q19	Do you choose to spend more time online over going out with others?	
Q20	Do you feel depressed, moody, or nervous when you are offline, which goes away when you are back online?	

The answers were of Likert scale-type as follows: 0 = Not Applicable; 1 = Rarely; 2 = Occasionally; 3 = Frequently; 4 = Often; 5 = Always.

**Table A2:** Compulsive internet use scale (CIUS).

Item	How Often...	Score
Q1	Do you find it difficult to stop using the internet when you are online?	
Q2	Do you continue to use the internet despite your intention to stop?	
Q3	Do others (e.g., partner, children, parents) say you should use the internet less?	
Q4	Do you prefer to use the internet instead of spending time with others (e.g., partner, children, parents)?	
Q5	Are you short of sleep because of the internet?	
Q6	Do you think about the internet, even when not online?	
Q7	Do you look forward to your next internet session?	
Q8	Do you think you should use the internet less often?	
Q9	Have you unsuccessfully tried to spend less time on the internet?	
Q10	Do you rush through your (home) work in order to go on the internet?	
Q11	Do you neglect your daily obligations (work, school, or family life) because you prefer to go on the internet?	
Q12	Do you go on the internet when you are feeling down?	
Q13	Do you use the internet to escape from your sorrows or get relief from negative feelings?	
Q14	Do you feel restless, frustrated, or irritated when you cannot use the internet?	

The answers were of Likert scale-type as follows: 0 = Never; 1 = Seldom; 2 = Sometimes; 3 = Often; 4 = Very often.
